# Association of *LIPC* polymorphisms with stroke risk in the Chinese population

**DOI:** 10.3389/fneur.2023.1095282

**Published:** 2023-05-18

**Authors:** Jiaxing Pan, Qingqing Zhuo, Xu Chen, Xuehong Huang, Shiqiang Shen, Qiu Yang, Jiawen Luo, Suiyan Wang, Tianbo Jin

**Affiliations:** ^1^Department of Neurology, People's Hospital of Wanning, Wanning, Hainan, China; ^2^Department of Nursing, People's Hospital of Wanning, Wanning, Hainan, China; ^3^College of Life Sciences, Northwest University, Xi'an, Shaanxi, China; ^4^Shaanxi Provincial Key Laboratory of Biotechnology, Northwest University, Xi'an, Shaanxi, China; ^5^Key Laboratory of Resource Biology and Biotechnology in Western China, Ministry of Education, Northwest University, Xi'an, Shaanxi, China

**Keywords:** LIPC, polymorphisms, stoke, susceptibility, Chinese population, case-control study

## Abstract

**Background:**

Stroke is a common cerebrovascular disease. The purpose of this study was to explore the association between *LIPC* single nucleotide polymorphisms (SNPs) and the risk of stroke in the Chinese population.

**Methods:**

This study recruited 710 stroke patients and 701 healthy controls. The four SNPs (rs690, rs6083, rs3829461, and rs6074) in *LIPC* were genotyped by the Agena MassARRAY. The correlation between *LIPC* polymorphisms and stroke risk was measured by odds ratio (OR) and 95% confidence interval (CI). In addition, multifactor dimensionality reduction (MDR) analysis was used to evaluate the impact of SNP–SNP interaction on stroke risk.

**Results:**

Overall analysis showed that rs690 was associated with an increased risk of stroke (T vs. G: OR = 1.19, 95% CI: 1.01–1.40, *p* = 0.041; additive: OR = 1.20, 95% CI: 1.01–1.42, *p* = 0.036). The stratified analysis revealed that rs690 was associated with an increased risk of stroke in subjects aged ≤ 64 years, male patients, and smokers, and rs6074 was associated with an increased risk of stroke in subjects aged > 64 years, male patients, drinkers, and non-smokers (*p* < 0.05). The results of the MDR analysis suggested the four-locus model as the most favorable model for assessing the risk of stroke. The analysis of clinical parameters of stroke patients showed that rs690 was correlated with platelet distribution width (PDW) (*p* = 0.014) and hematocrit levels (*p* = 0.004), and rs6074 was correlated with low-density lipoprotein cholesterol (LDL-C) level (*p* = 0.033). Furthermore, bioinformatics analysis results demonstrated that the expression levels of *LIPC* and its related genes (*APOB, CETP, PNPLA2*, and *LMF1*) were significantly different between the control and stroke groups (*p* < 0.05), and *LIPC*-related proteins were mainly related to lipid metabolism.

**Conclusion:**

This study indicated that rs690 and rs6074 in *LIPC* were significantly associated with increased risk of stroke in the Chinese population, possibly by regulating the levels of PDW, HCT, and LDL-C.

## Introduction

Stroke is manifested by neurological deficits caused by impaired blood circulation in the brain. It is mainly described as a brain disease with symptoms such as sudden faintness, hemiplegia, numbness of the limbs, and tilted mouth and tongue. Although medical treatment has made great progress in recent years, the incidence of stroke is still increasing due to factors such as population aging and changes in people's living habits, making it the world's second-leading cause of death and the main cause of disability (1). Notably, China is facing the largest burden of stroke (2) in recent years, with the incidence and prevalence of stroke still increasing, especially in rural areas (3). Deaths from stroke in China account for almost one-third of the total stroke deaths in the world (4). Therefore, research studies on stroke are particularly necessary.

A large number of studies have indicated that genetic factors play an important role in the pathogenesis of stroke (5). Moreover, hypertension, diabetes, atrial fibrillation, smoking, hyperlipidemia, and obesity are also studied to be risk factors for stroke (6). Lipase C (LIPC) is located on the long arm of chromosome 15 in band 21.3 and is mainly responsible for encoding lipase C. The dual function of the *LIPC* gene, namely, triglyceride hydrolase and ligand/bridging factor, is mainly used for receptor-mediated lipoprotein uptake. Common variants in *LIPC* have been shown to be associated with plasma lipid levels (7, 8). A previous study has demonstrated an association between the polymorphism (-514 C > T) in *LIPC* and type 2 diabetes risk, which is modified by the high-density lipoprotein cholesterol (HDL-C) level (9). *LIPC* variant rs2070895 has been found to be associated with carotid atherosclerosis (10) and hypertension (11). Moreover, *LIPC* variants (rs2043085 and rs1532085) have been observed to be significantly associated with body mass index (BMI), waist circumference (WC), lipid accumulation product, visceral adiposity index, and triglyceride glucose (TyG) index-related parameters (12). However, there are very few studies on the correlation between *LIPC* polymorphisms and the risk of stroke.

Therefore, this study was undertaken to detect the association between *LIPC* polymorphisms and the risk of stroke from 710 patients with stroke and 701 healthy controls in the Chinese population. The results of this study will provide a certain scientific theoretical basis for early screening, prevention, and diagnosis of stroke in the Chinese population.

## Materials and methods

### Participants

We recruited 710 stroke patients (430 male patients and 280 female patients) from the People's Hospital of Wanning who were admitted to the hospital within 72 h after the onset of symptoms. All patients with focal neurological deficit symptoms persisting for more than 24 h were examined by two professional stroke neurologists and diagnosed by brain computed tomography (CT), magnetic resonance imaging (MRI), cerebrovascular angiography, and lumbar puncture (13). This study excluded patients with transient ischemic attack, lacunar infarction, familial stroke, severe heart, kidney, liver, endocrine, and bone diseases, and cancers. A total of 701 healthy participants (424 male patients and 277 female patients) were selected as the control group from the health examination center of the People's Hospital of Wanning during the same period as the cases. All control individuals had no family history of stroke, severe heart, kidney, liver, endocrine and bone diseases, cancers, and immune-related diseases. The gender and age of the control group were matched with the patients in the case group. The basic information of all subjects, including age, sex, smoking status, drinking status, and clinical test indicators, were collected from questionnaires and clinical data. This study was approved by the Ethics Committee of the People's Hospital of Wanning and conformed to the Declaration of Helsinki. All subjects were informed of the purpose, significance, and experimental process of the study before the experiment. This study was conducted after obtaining informed consent from subjects.

### SNP selection and genotyping

The four *LIPC* polymorphisms (rs690, rs6083, rs3829461, and rs6074) were randomly selected in this study according to previous articles (7, 14, 15). All SNPs had a minor allele frequency (MAF) > 0.05 in the Chinese Han Beijing (CHB) population from the 1000 Genomes Project (http://www.internationalgenome.org/). The functions of candidate SNPs were predicted using the NCBI dbSNP (http://www.ncbi.nlm.nih.gov/projects/SNP) and HaploReg v4.1 databases (https://pubs.broadinstitute.org/mammals/haploreg/haploreg.php) (16). The results showed that the four SNPs in *LIPC* were both synonymous and coding sequence variants. Peripheral venous blood (5 mL) from each subject was collected for DNA extraction according to the instructions of the whole blood genomic DNA extraction kit (GoldMag Co. Ltd. Xi'an, China) (17). The DNA concentration and purity were determined by spectrometry (NanoDrop 2000 spectrophotometer, Thermo Scientific, USA). In this study, the Agena Bioscience Assay Design Suite was performed (3.1) to design primers (polymerase chain reaction primers and unique base extension primer) for the four SNPs in *LIPC*. These primers were synthesized by Bioengineering (Shanghai Co., Ltd). The Agena MassARRAY platform (Agena Bioscience, San Diego, CA, USA) was used to genotype the four SNPs. Ultimately, the Agena Typer Software (version 4.0, Agena Bioscience, USA) was used for data analysis and processing (18).

### Statistical analysis

The differences in gender and drinking and smoking status between cases and controls were assessed using the χ^2^-test. The differences in age and clinical indicators between the two groups were analyzed by the independent samples *t*-test. PLINK software (version 1.0.7) was used to calculate odds ratios (ORs) and 95% confidence intervals (CIs) by logistic regression analysis to measure the association between *LIPC* polymorphisms and stroke risk in the Chinese population under genetic models (allele, genotype, dominant, and recessive and additive models) adjusted by confounding factors. In order to control the influence of confounding factors on the results, we conducted the analyses stratified by age, sex, and smoking and drinking status (19). Data analysis was performed using SPSS version 20.0 (SPSS Inc., Chicago, IL, USA). A *P*-value of < 0.05 was defined as statistical significance. The G^*^Power 3.1.9.7 software was used to calculate the sample size and statistical power of this study, (20). Parameter is tails = 2, effect size = 0.2, α = 0.05, power = 0.964, and allocation ratio =1. Multifactor dimensionality reduction (MDR) analysis was performed to evaluate the impact of SNP–SNP interaction on the risk of stroke in the Chinese population (21).

### Bioinformatics analysis

*LIPC*-related protein analysis was conducted through the STRING online database (https://cn.string-db.org/). We downloaded the GSE16561 dataset (39 cases and 24 controls) from the GEO database. First, standardize and log the original data. Second, select matching samples (24 cases and 24 controls). Finally, the “Limma” package in R was used to analyze the difference in the expression of *LIPC* and its related genes in the control and stroke groups. Furthermore, the biological process of *LIPC*-related protein–protein interaction was analyzed through the Sangerbox database (http://sangerbox.com/home.html).

## Results

### Subject characteristics

In this study, a total of 710 stroke patients and 701 controls were recruited to explore the correlation between *LIPC* polymorphisms and the risk of stroke in the Chinese population. In the case group, there were 360 patients older than 64 years and 270 patients younger than or equal to 64 years. In the control group, 271 individuals aged greater than 64 years and 430 individuals aged less than or equal to 64 years. The average ages of cases and controls were 64.44 ± 10.738 years and 64.12 ± 5.697 years, respectively. There were 854 male patients and 557 female patients in all subjects. Detailed information on subjects, such as smoking and drinking status, is shown in Table 1. There were no significant differences in the distribution of age, gender, and drinking and smoking status between the case and control groups (*p* > 0.05).

**Table 1:** Demographic characteristics of cases and controls in this study.

**Characteristics**		**Cases (*N =* 710)**	**Controls (*N =* 701)**	***P*-value**
Age	Mean ± SD	64.44 ± 10.738	64.12 ± 5.697	0.486^a^
	> 64 years	350 (49.30%)	271 (38.7%)	
	≤ 64 years	360 (50.70%)	430 (61.3%)	
Sex	Male	430 (60.6%)	424 (60.5%)	0.976^b^
	Female	280 (39.4%)	277 (39.5%)	
Smoking status	Yes	352 (49.6%)	347 (49.5%)	0.977^b^
	No	358 (50.4%)	354 (50.5%)	
Drinking status	Yes	356 (50.1%)	355 (50.6%)	0.851^b^
	No	354 (49.9%)	346 (49.4%)	

^a^Independent samples t-test; ^b^Pearson's chi-squared test; P < 0.05 indicates statistical significance.

### The overall analysis of the relationship between *LIPC* polymorphisms and stroke risk

The basic information, including the chromosome number, physical location, and alleles of the candidate SNPs, is presented in Table 2. All genotype distribution of four SNPs in *LIPC* (rs690, rs6083, rs3829461, and rs6074) met HWE (*p* > 0.05), indicating that subjects in the study were in a state of genetic balance, and the genotyping results were reliable. The frequency distribution of alleles T and G of rs690 was significantly different between the case and control groups. Compared with the allele G of rs690, allele T was significantly associated with an increased risk of stroke (OR = 1.19, 95% CI: 1.01–1.40, *p* = 0.041) (Table 2). In the additive model, rs690 was found to be associated with an increased risk of stroke (OR = 1.20, 95% CI: 1.01–1.42, *p* = 0.036) (Table 3). However, no statistical significance was found between the genotypes of the three SNPs (rs6083, rs3829461, and rs6074) in *LIPC* and the risk of stroke (*p* > 0.05).

**Table 2:** Distribution of allele of SNPs in *LIPC* and their association with stroke risk.

**SNP-ID**	**Chr**	**Position**	**Allele**	**MAF**		***p*-HWE**	**OR (95% CI)**	***p***
			**A/B**	**Case**	**Control**			
rs690	15	58542542	T/G	0.292	0.258	0.236	1.19 (1.01–1.40)	**0.041**
rs6083	15	58545811	A/G	0.167	0.153	0.661	1.11 (0.91–1.36)	0.301
rs3829461	15	58560910	A/G	0.062	0.068	0.764	0.89 (0.66–1.20)	0.455
rs6074	15	58568764	A/C	0.285	0.265	0.439	1.11 (0.94–1.30)	0.237

SNP, single nucleotide polymorphism; alleles A/B, minor/major alleles; MAF, minor allele frequency; OR, odds ratio; CI, confidence interval; HWE, Hardy–Weinberg equilibrium; P-values were calculated with Pearson's χ^2^ tests; P < 0.05 and bold values indicate statistical significance. Bonferroni p < 0.0125 (0.05/4) indicates statistical significance.

**Table 3:** Relationships between *LIPC* polymorphisms and stroke risk under multiple genetic models.

**SNP-ID**	**Models**	**Genotypes**	**Controls**	**Cases**	**OR (95% CI)**	***P*-value**
rs690	Codominant	G/G	379 (54.2%)	349 (49.2%)	1	
		G/T	280 (40.1%)	306 (43.2%)	1.19 (0.96–1.47)	0.121
		T/T	40 (5.7%)	54 (7.6%)	1.46 (0.95–2.26)	0.085
	Dominant	G/G	379 (54.2%)	349 (49.2%)	1	
		G/T-T/T	320 (45.8%)	360 (50.8%)	1.22 (0.99–1.51)	0.060
	Recessive	G/G-G/T	659 (94.3%)	655 (92.4%)	1	
		T/T	40 (5.7%)	54 (7.6%)	1.36 (0.89–2.07)	0.159
	Additive	—	—	—	1.20 (1.01–1.42)	**0.036**
rs6083	Codominant	G/G	504 (72%)	487 (68.7%)	1	
		G/A	178 (25.4%)	207 (29.2%)	1.20 (0.95–1.52)	0.123
		A/A	18 (2.6%)	15 (2.1%)	0.86 (0.43–1.72)	0.666
	Dominant	G/G	504 (72%)	487 (68.7%)	1	
		G/A-A/A	196 (28%)	222 (31.3%)	1.17 (0.93–1.47)	0.175
	Recessive	G/G-G/A	682 (97.4%)	694 (97.9%)	1	
		A/A	18 (2.6%)	15 (2.1%)	0.81 (0.41–1.63)	0.562
	Additive	—	—	—	1.11 (0.91–1.37)	0.301
rs3829461	Codominant	G/G	607 (86.6%)	623 (88.1%)	1	
		A/G	92 (13.1%)	81 (11.5%)	0.86 (0.62–1.18)	0.347
		A/A	2 (0.3%)	3 (0.4%)	1.45 (0.24–8.74)	0.682
	Dominant	G/G	607 (86.6%)	623 (88.1%)	1	
		A/G-A/A	94 (13.4%)	84 (11.9%)	0.87 (0.64–1.19)	0.389
	Recessive	G/G-A/G	699 (99.7%)	704 (99.6%)	1	
		A/A	2 (0.3%)	3 (0.4%)	1.48 (0.25–8.90)	0.667
	Additive	—	—	—	0.89 (0.66–1.20)	0.452
rs6074	Codominant	C/C	383 (54.6%)	381 (53.7%)	1	
		C/A	265 (37.8%)	254 (35.8%)	0.96 (0.77–1.20)	0.737
		A/A	53 (7.6%)	75 (10.6%)	1.42 (0.97–2.08)	0.069
	Dominant	C/C	383 (54.6%)	381 (53.7%)	1	
		C/A-A/A	318 (45.4%)	329 (46.3%)	1.04 (0.84–1.28)	0.720
	Recessive	C/C-C/A	648 (92.4%)	635 (89.4%)	1	
		A/A	53 (7.6%)	75 (10.6%)	1.45 (1.00–2.09)	0.050
	Additive	—	—	—	1.10 (0.94–1.29)	0.257

SNP, single nucleotide polymorphism; OR, odds ratio; 95% CI, 95% confidence interval; OR (95% CI) values were calculated by logistic regression analysis adjusted by age, sex, smoking, and drinking. P < 0.05 and bold values indicate statistical significance. Bonferroni p < 0.003125 (0.05/4/4) indicates statistical significance.

### Stratified analysis of the relationship between *LIPC* polymorphisms and stroke risk

This study further clarified the correlation between *LIPC* polymorphisms and the risk of stroke in the Chinese population after stratification analysis by age, gender, and smoking and drinking status. The results of the age-stratified analysis showed that rs690 was found to be associated with an increased risk of stroke in subjects aged ≤ 64 years (T vs. G: OR = 1.30, 95% CI: 1.04–1.63, *p* = 0.020; additive: OR = 1.31, 95% CI: 1.01–1.70, *p* = 0.044). In the subgroup aged < 64 years, rs6074 was found to be associated with an increased risk of stroke (AA vs. CC: OR = 1.98, 95% CI: 1.07–3.65, *p* = 0.030 AA vs. AC-CC: OR = 1.84, 95% CI: 1.01–3.34, *p* = 0.046; additive: OR = 1.32, 95% CI: 1.02–1.71, *p* = 0.034) (Table 4). Gender-stratified analysis revealed that rs690 was associated with an increased risk of stroke in male patients under the allele (T vs. G: OR = 1.25, 95% CI: 1.01–1.55, *p* = 0.042), homozygote (TT vs. GG: OR = 1.82, 95% CI: 1.03–3.23, *p* = 0.040), and additive (OR = 1.26, 95% CI: 1.01–1.57, *p* = 0.039) models. Compared with genotypes A/C and C/C, male patients with genotype A/A of rs6074 were more susceptible to stroke (OR = 1.76, 95% CI: 1.06–2.93, *p* = 0.029; recessive: OR = 1.76, 95% CI: 1.07–2.89, *p* = 0.025) (Table 4). Nevertheless, no association between four SNPs in *LIPC* and the susceptibility to stroke in female patients was detected.

**Table 4:** Relationships of *LIPC* polymorphisms with stroke risk stratified by gender and age.

**SNP-ID**	**Models**	**Genotypes**	**Age** > **64**		**Age** ≤ **64**		**Males**		**Females**	
			**OR (95% CI)**	***P*** **-value**	**OR (95% CI)**	***P*** **-value**	**OR (95% CI)**	***P*** **-value**	**OR (95% CI)**	***P*** **-value**
rs690	Allele	G	1		1		1		1	
		T	1.04 (0.81–1.33)	0.778	1.30 (1.04–1.63)	**0.020**	1.25 (1.01–1.55)	**0.042**	1.11 (0.85–1.44)	0.451
	Codominant	G/G	1		1		1		1	
		T/G	1.01 (0.71–1.43)	0.966	1.31 (0.94–1.81)	0.112	1.18 (0.89–1.56)	0.250	1.19 (0.84–1.68)	0.326
		T/T	1.32 (0.69–2.52)	0.394	1.73 (0.86–3.47)	0.122	1.82 (1.03–3.23)	**0.040**	1.06 (0.54–2.08)	0.874
	Dominant	G/G	1		1		1		1	
		T/G-T/T	1.05 (0.75–1.47)	0.769	1.35 (0.98–1.85)	0.064	1.25 (0.96–1.64)	0.104	1.17 (0.84–1.63)	0.355
	Recessive	G/G-T/G	1		1		1		1	
		T/T	1.32 (0.71–2.46)	0.384	1.54 (0.78–3.04)	0.214	1.70 (0.97–2.97)	0.064	0.98 (0.50–1.89)	0.942
	Additive	-	1.09 (0.83–1.41)	0.545	1.31 (1.01–1.70)	**0.044**	1.26 (1.01–1.57)	**0.039**	1.1 0(0.84–1.45)	0.473
rs6074	Allele	C	1		1		1		1	
		A	1.28 (0.99–1.65)	0.058	0.99 (0.79–1.24)	0.930	1.19 (0.96–1.48)	0.104	0.99 (0.76–1.28)	0.910
	Codominant	C/C	1		1		1		1	
		A/C	1.20 (0.84–1.71)	0.323	0.89 (0.64–1.26)	0.516	1.00 (0.75–1.33)	0.988	0.90 (0.63–1.29)	0.577
		A/A	1.98 (1.07–3.65)	**0.030**	1.48 (0.84–2.58)	0.172	1.76 (1.06–2.93)	**0.029**	1.08 (0.61–1.92)	0.799
	Dominant	C/C	1		1		1		1	
		A/A-A/C	1.32 (0.94–1.85)	0.103	0.99 (0.72–1.36)	0.944	1.11 (0.85–1.46)	0.438	0.94 (0.67–1.31)	0.706
	Recessive	A/C-C/C	1		1		1		1	
		A/A	1.84 (1.01–3.34)	**0.046**	1.55 (0.90–2.65)	0.115	1.76 (1.07–2.89)	**0.025**	1.12 (0.64–1.96)	0.683
	Additive	-	1.32 (1.02–1.71)	**0.034**	1.08 (0.85–1.38)	0.521	1.18 (0.96–1.45)	0.116	0.99 (0.77–1.27)	0.920

SNP, single nucleotide polymorphism; OR, odds ratio; 95% CI, 95% confidence interval; OR (95% CI) values were calculated by logistic regression analysis adjusted by age, sex, smoking, and drinking. P < 0.05 and bold values indicate statistical significance. Bonferroni p < 0.0025 (0.05/4/5) indicates statistical significance.

After stratification by drinking status, the analysis indicated that rs6074 was associated with an increased risk of stroke in drinkers under the recessive model (AA vs. AC-CC: OR = 1.64, 95% CI: 1.00–2.68 *p* = 0.049). However, no SNP was observed to be related to stroke risk in the non-drinking subgroup (*p* > 0.05) (Table 5). In the analysis stratified by smoking status, we found that rs690 was associated with an increased risk of stroke in smokers under the allele (OR = 1.29, 95% CI: 1.02–1.63, *p* = 0.037), dominant (OR = 1.37, 95% CI: 1.02–1.85, *p* = 0.038), and additive (OR = 1.28, 95% CI: 1.00–1.63, *p* = 0.048) models. In the non-smoking subgroup, rs6074 was found to be associated with an increased risk of stroke (A vs. C: OR = 1.33, 95% CI: 1.05–1.68, *p* = 0.016; AA vs. CC: OR = 2.04, 95% CI: 1.20–3.46, *p* = 0.008; additive: OR = 1.30, 95% CI: 1.04–1.63, *p* = 0.020).

**Table 5:** Relationships of *LIPC* gene polymorphisms with the risk of stroke stratified by smoking and drinking.

**SNP-ID**	**Models**	**Genotypes**	**Drinking**		**No-drinking**		**Smoking**		**No-smoking**	
			**OR (95%CI)**	***P*** **-value**	**OR (95%CI)**	***P*** **-value**	**OR (95%CI)**	***P*** **-value**	**OR (95%CI)**	***P*** **-value**
rs690	Allele	G	1		1		1		1	
		T	1.22 (0.96–1.53)	0.103	1.16 (0.92–1.47)	0.207	1.29 (1.02–1.63)	**0.037**	1.10 (0.87–1.39)	0.413
	Codominant	G/G	1		1		1		1	
		T/G	1.14 (0.84–1.55)	0.410	1.25 (0.92–1.71)	0.156	1.36 (1.00–1.86)	0.053	1.01 (0.75–1.38)	0.934
		T/T	1.73 (0.92–3.22)	0.087	1.22 (0.66–2.25)	0.517	1.45 (0.79–2.66)	0.235	1.45 (0.78–2.70)	0.245
	Dominant	G/G	1		1		1		1	
		T/G-T/T	1.20 (0.9–1.62)	0.220	1.25 (0.93–1.68)	0.145	1.37 (1.02–1.85)	**0.038**	1.06 (0.79–1.43)	0.690
	Recessive	G/G-T/G	1		1		1		1	
		T/T	1.63 (0.89–3.00)	0.117	1.11 (0.61–2.01)	0.737	1.26 (0.70–2.29)	0.442	1.44 (0.78–2.65)	0.240
	Additive	-	1.22 (0.96–1.55)	0.103	1.18 (0.92–1.50)	0.189	1.28 (1.00–1.63)	**0.048**	1.10 (0.87–1.40)	0.425
rs6074	Allele	C	1		1		1		1	
		A	1.18 (0.94–1.48)	0.155	1.03 (0.81–1.31)	0.811	0.91 (0.72–1.16)	0.444	1.33 (1.05–1.68)	**0.016**
	Codominant	C/C	1		1		1		1	
		A/C	0.96 (0.70–1.32)	0.822	0.96 (0.70–1.32)	0.804	0.84 (0.61–1.15)	0.267	1.12 (0.81–1.53)	0.496
		A/A	1.61 (0.97–2.69)	0.065	1.18 (0.66–2.10)	0.580	0.92 (0.52–1.61)	0.762	2.04 (1.20–3.46)	**0.008**
	Dominant	C/C	1		1		1		1	
		A/A-A/C	1.08 (0.80–1.45)	0.621	1.00 (0.74–1.34)	0.974	0.85 (0.63–1.15)	0.287	1.27 (0.94–1.70)	0.119
	Recessive	A/C-C/C	1		1		1		1	
		A/A	1.64 (1.00–2.68)	**0.049**	1.20 (0.68–2.10)	0.535	0.99 (0.57–1.70)	0.962	1.95 (1.17–3.26)	**0.011**
	Additive	-	1.15 (0.93–1.44)	0.204	1.03 (0.81–1.30)	0.815	0.90 (0.72–1.14)	0.397	1.30 (1.04–1.63)	**0.020**

SNP, single nucleotide polymorphism; OR, odds ratio; 95% CI, 95% confidence interval; OR (95% CI) values were calculated by logistic regression analysis adjusted by age, sex, smoking, or drinking. P < 0.05 and bold values indicate statistical significance. Bonferroni p < 0.0025 (0.05/4/5) indicates statistical significance.

### The relationship between *LIPC* polymorphisms and clinical indicators of stroke patients

**Figure 1:**
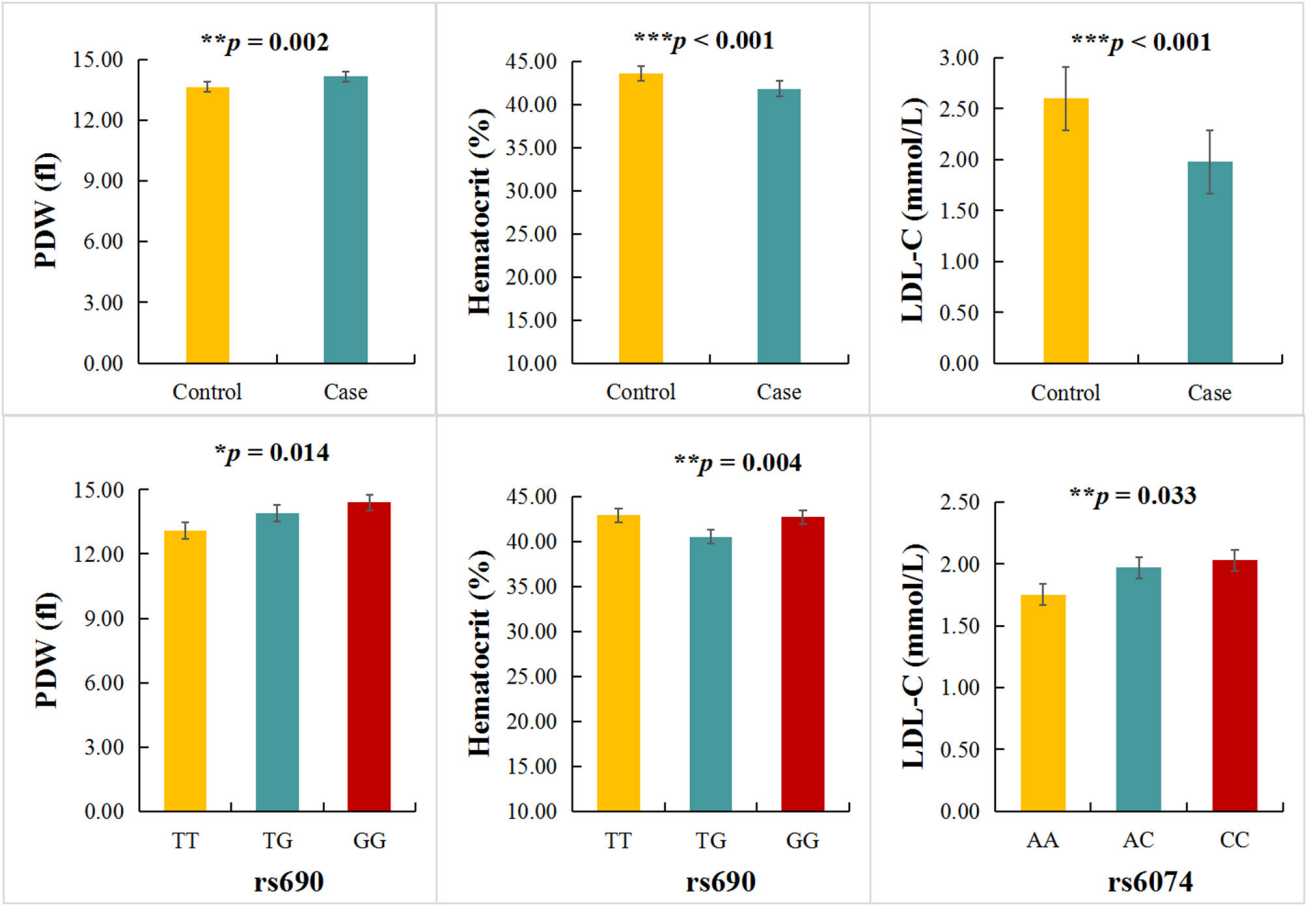
Association between *LIPC* polymorphisms and clinical indicators. ^*^p < 0.05, ^**^p < 0.01, ^***^p < 0.001.

The relationship between *LIPC* polymorphisms and 42 clinical parameters of stroke patients was also investigated in the study (Supplementary Table 1). We found a significant difference in platelet distribution width (PDW), hematocrit (HCT), and low-density lipoprotein cholesterol (LDL-C) levels between cases and controls (*p* < 0.05). The TT, TG, and GG genotypes of rs690 were significantly correlated with PDW (*p* = 0.014) and HCT (*p* = 0.004) levels (Figure 1). The TT, TG, and GG genotypes of rs6074 were significantly correlated with the level of LDL-C (*p* = 0.033) (Supplementary Table 1 and Figure 1).

### MDR analysis of the effect of SNP-SNP interactions on stroke risk

**Figure 2:**
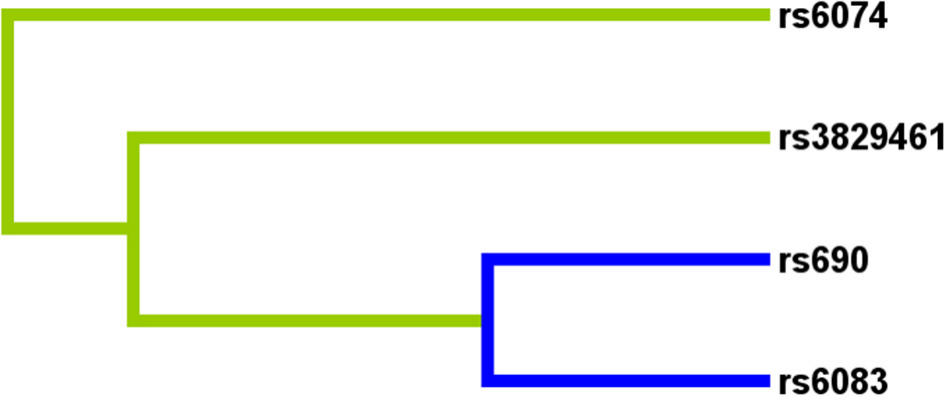
Dendogram of the effect of *LIPC* SNP-SNP interaction on stroke risk.

Multifactor dimensionality reduction analysis was performed to further explore the impact of *LIPC* SNP-SNP interaction on stroke risk. Figure 2 shows that rs690, rs6083, rs3829461, and rs6074 in *LIPC* had antagonistic effects on stroke risk. In the MDR analysis, by comparing the parameters of cross-validation consistency (CVC), OR, 95% CI, *p*-value, training balanced accuracy (Bal. Acc.), and testing balanced accuracy, we found that the four-locus model consisting of four SNPs (rs690, rs6083, rs3829461, and rs6074) was the best one to assess the risk of stroke (CVC: 10/10; Testing Bal. Acc = 0.519; OR = 1.66, 95% CI: 1.32–2.07, *p* = 0.0001, Table 6).

**Table 6:** Impact of SNP-SNP interaction on stroke risk.

**Model**	**Training Bal. Acc**	**Testing Bal. Acc**	**CVC**	**OR (95% CI)**	***P*-value**
rs690	0.527	0.501	8/10	1.25 (1.00–1.56)	0.0511
rs690,rs6074	0.538	0.501	7/10	1.38 (1.10–1.74)	**0.0051**
rs690,rs3829461,rs6074	0.550	0.506	5/10	1.51 (1.20–1.89)	**0.0003**
rs690,rs6083,rs3829461,rs6074	0.563	0.519	10/10	1.66 (1.32–2.07)	**0.0001**

MDR, multifactor dimensionality reduction; Bal. Acc., balanced accuracy; CVC, cross-validation consistency. P < 0.05 and bold values indicate statistical significance.

### Bioinformatics analysis

**Figure 3:**
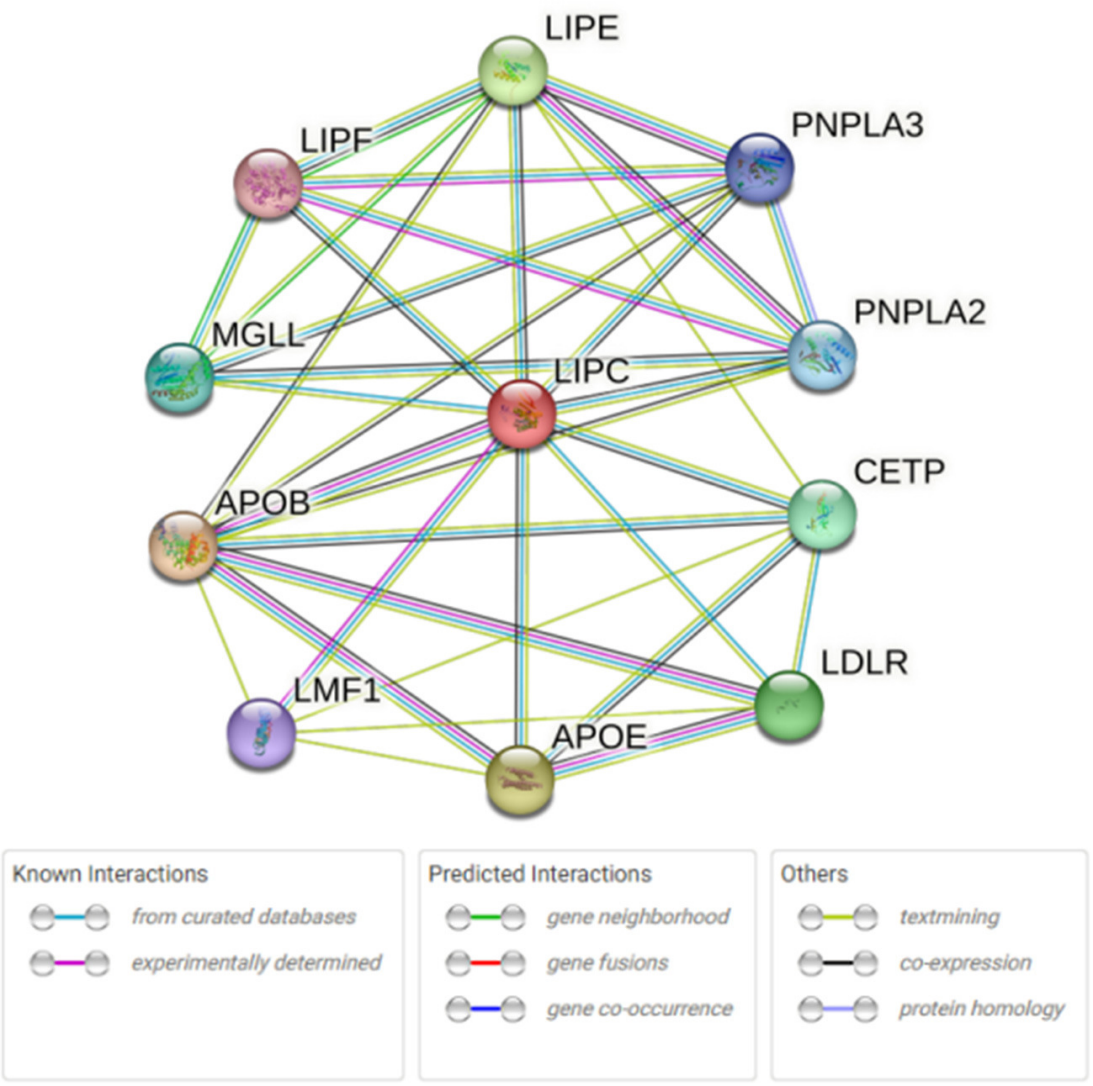
Main protein that interacts with *LIPC*.

**Figure 4:**
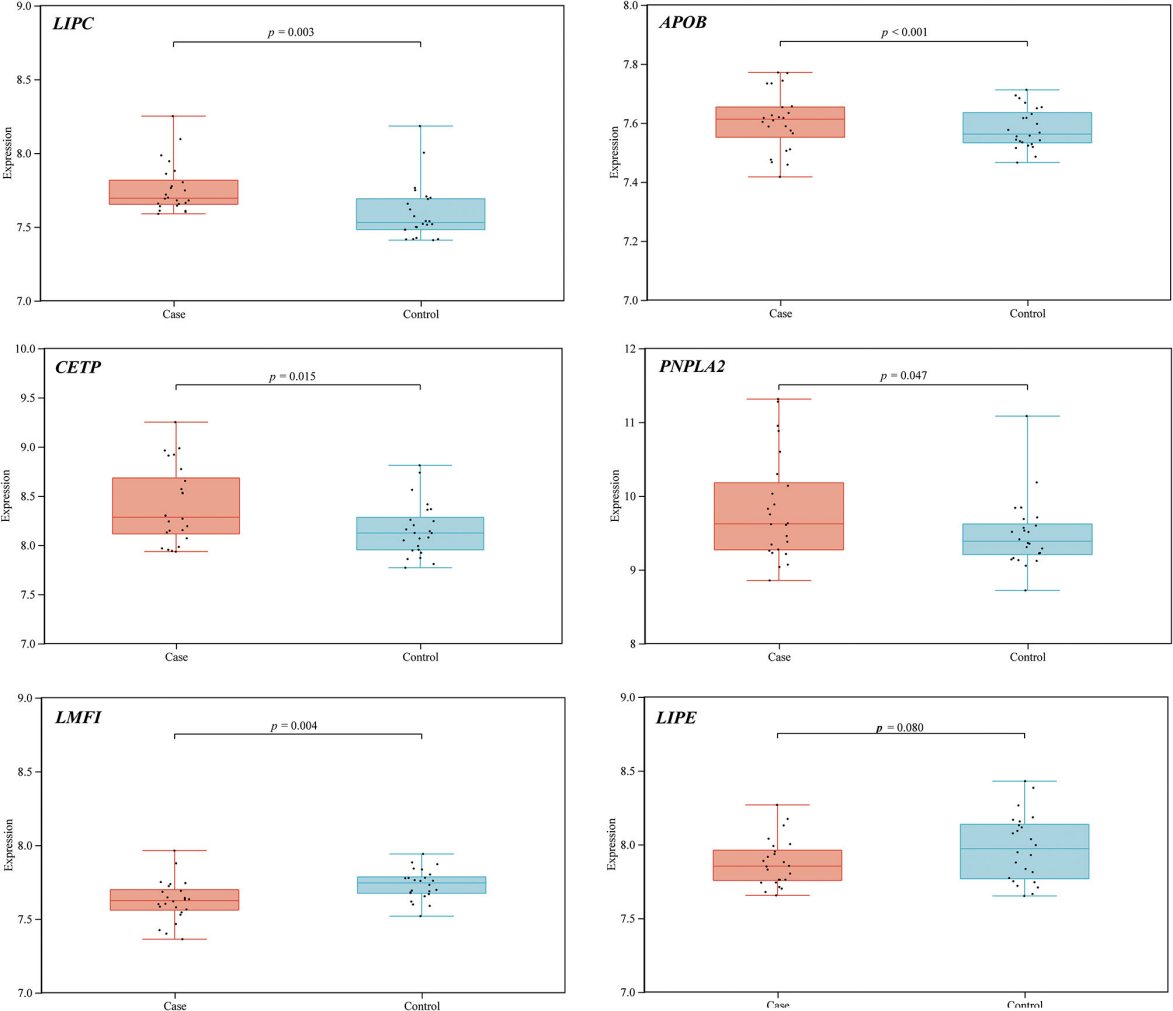
Expression of *LIPC* and its related genes in the control and stroke groups.

**Figure 5:**
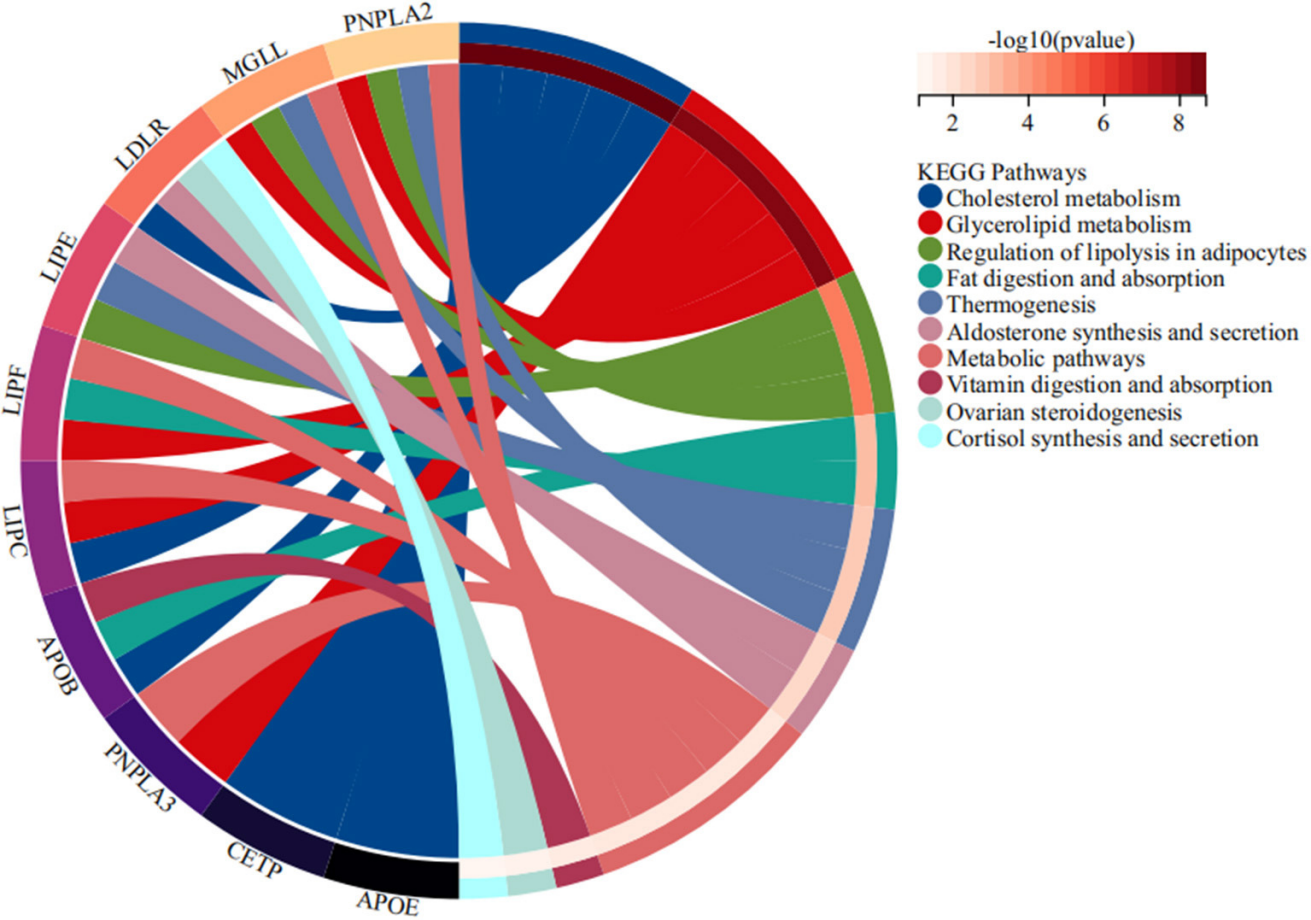
Main biological process of the main protein–protein interactions related to *LIPC*.

STRING database analysis results showed that there were nine main proteins related to *LIPC*, namely, APOB, APOE, CETP, LDLR, LIPE, LIPF, MGLL, PNPLA3, PNPLA2, and LMF1 (Figure 3). Based on the Gene Expression Omnibus (GEO) database analysis, significant differences in the expression levels of *LIPC, APOB, CETP, PNPLA2*, and *LMF1* between the control and stroke groups were observed (Supplementary Table 2 and Figure 4). Furthermore, the Sangerbox analysis results showed that the biological processes of the main protein–protein interactions related to *LIPC* mainly involved the metabolism of cholesterol and glycerolipid pathways (Figure 5).

## Discussion

This is the first study to analyze the relationship between *LIPC* polymorphisms and the risk of stroke in the Chinese population. In the overall analysis, we found that rs690 was associated with an increased risk of stroke in the Chinese population. The stratified analysis revealed that rs690 was associated with an increased risk of stroke in subjects aged >64 years, male subjects, and smokers. In addition, s6074 was associated with an increased risk of stroke in subjects aged ≤ 64 years, male subjects, non-smokers, and drinkers. In addition, we found that rs690 was associated with PDW and HCT levels, and rs6074 is related to the LDL-C level. MDR analysis results demonstrated that the four-locus model was the best model for stroke risk assessment.

*LIPC* encodes the hepatic lipase. A whole-exome sequencing study has revealed that the abnormal combination of the AA genotype of rs17269397 in *LIPC* and cholesterol levels can affect the risk of coronary artery disease (CAD) (22). A study using the meta-analysis approach has shown that rs2070895 in *LIPC* was associated with an increased risk of hypertension (11). Previous studies have demonstrated that *LIPC* plays a vital role in the pathogenesis of cardiovascular and cerebrovascular diseases. In addition, it has been elucidated that *LIPC* exhibits a gene-level association with myocardial infarction (23). This study was the first to reveal that *LIPC* polymorphisms were associated with the risk of stroke in the Chinese population.

It has been reported that age, gender, alcohol consumption, and smoking are important risk factors for stroke (24). More precisely, the main driver for increased stroke prevalence is the aging of the population (25, 26). The incidence of stroke is higher in male patients, and also male patients have their first stroke earlier than female patients. However, female patients have more severe strokes than male patients (27, 28). Alcohol consumption has complex effects on cardiovascular health, and views on the associations between alcohol consumption and stroke risk remain controversial (29, 30). Cigarette smoking is a well-established risk factor for all forms of stroke. Continued smoking after a stroke is associated with a high risk of stroke recurrence and other cardiovascular diseases (31). Our research has shown that rs690 and rs6074 in *LIPC* were associated with an increased risk of stroke in male patients. These two loci were also associated with an increased risk of stroke in different age subgroups ( ≤ 64 years and < 64 years, respectively). Furthermore, the T allele of rs690 was associated with an increased risk of stroke in smokers. The allele A of rs6074 was associated with an increased risk of stroke in non-smokers and alcohol drinkers. Hence, we speculated that the impacts of age, gender, smoking, and drinking on the susceptibility to stroke were stronger than the combination of rs690 and rs6074.

Studies have shown that the combination of rs690 in *LIPC* and other SNPs in *LIPC* can affect the bioavailability of dietary cholesterol in healthy adult male patients (32) and is associated with the HDL-C level (7). Our research also showed that rs690 was associated with the PDW and HCT levels of stroke patients instead of HDL-C. PDW was an important risk factor for stroke in atrial fibrillation patients (33). Several large-scale observational studies have pointed out an increased risk of stroke in individuals with elevated HCT (34). Therefore, we speculated that rs690 in *LIPC* modified the risk of stroke by modulating PDW and/or HCT levels. Among South Indian subjects without diabetes, the CA genotype of rs6074 has been found to be associated with a low level of HDL-C (35), suggesting that rs6074 is related to the LDL-C level rather than HDL-C. Previous study has found that a very low LDL-C level is associated with high risks of all-cause and stroke mortality (36), thus, the AA genotype of rs6074 was associated with an increased risk of stroke by regulating the LDL-C level. *LIPC* polymorphism rs6083 has been found to be associated with cholesterol, triglyceride, LDL-C (14), and HDL-C levels (7). However, little is known about the association between rs6083 and the clinical parameters of stroke patients.

As genetic polymorphisms worked jointly on disease risk, MDR analysis was conducted in this study to further explore the impact of SNP–SNP interactions on the risk of stroke, indicating that SNP–SNP interactions demonstrate an antagonistic effect on the risk of stroke, and the combination of the four sites is the best risk assessment model for stroke. A study has exhibited an association between abnormal lipid metabolism and stroke (37). Our bioinformatics analysis results indicated that the expression levels of *LIPC* and its related genes (*APOB, CETP, PNPLA2*, and *LMF1*) showed significant differences between stroke and control groups. The main *LIPC*-related protein–protein interactions were connected with lipid metabolism, which is expected to provide a new direction for investigating the effect of *LIPC* on stroke risk.

There are several limitations to this study. First, this study is the first to explore the relationship between *LIPC* polymorphisms and stroke susceptibility in the Chinese population. To further verify the results, a large sample will be collected in the following studies. Second, the subjects in this study came from the same hospital during the same period, and the geographic limitations in sample selection should not be overlooked. Third, we only predicted the signal pathway that *LIPC* might participate in through the databases, and the impact of SNPs on the *LIPC* gene mRNA level and the function of *LIPC* in stroke have not been explored in this study.

## Conclusion

These findings revealed that *LIPC* polymorphisms (rs690 and rs6074) were associated with an increased risk of stroke in the Chinese population, which may modify stroke risk by regulating PDW, HCT, and LDL-C levels, respectively. These results provide a novel direction for the early screening and prevention of stroke in a high-risk population in China.

## Data availability statement

The raw data supporting the conclusions of this article will be made available by the authors, without undue reservation.

## Ethics statement

The studies involving human participants were reviewed and approved by the Ethics Committee of People's Hospital of Wanning and in compliance with the Declaration of Helsinki. The patients/participants provided their written informed consent to participate in this study.

## Author contributions

SW and TJ: conceived and designed the experiments and drafted the article and/or revised it critically for important content. JP and QZ: performed the experiments. XC and XH: analyzed the data. SS: responsible for reagents, materials, and analysis tools. QY and JL: prepared the figures and/or tables. All authors have read and approved the manuscript.
